# Molecular detection and characterization of *Cryptosporidium* spp., *Giardia duodenalis*, and *Enterocytozoon bieneusi* infections in dromedary camels (*Camelus dromedaries*) in Egypt

**DOI:** 10.3389/fvets.2023.1139388

**Published:** 2023-04-20

**Authors:** Ehab Kotb Elmahallawy, Pamela C. Köster, Alejandro Dashti, Samia Qasem Alghamdi, Amira Saleh, Ahmed Gareh, Barakat M. Alrashdi, Carolina Hernández-Castro, Begoña Bailo, Maha S. Lokman, Eman A. A. Hassanen, David González-Barrio, David Carmena

**Affiliations:** ^1^Department of Zoonoses, Faculty of Veterinary Medicine, Sohag University, Sohag, Egypt; ^2^Parasitology Reference and Research Laboratory, National Centre for Microbiology, Madrid, Spain; ^3^Department of Biology, Faculty of Science, Al-Baha University, Alaqiq, Al-Baha Province, Saudi Arabia; ^4^Department of Medical Parasitology, Faculty of Medicine, Zagazig University, Zagazig, Egypt; ^5^Department of Parasitology, Faculty of Veterinary Medicine, Aswan University, Aswan, Egypt; ^6^Biology Department, College of Science, Jouf University, Sakaka, Saudi Arabia; ^7^Parasitology Group, Faculty of Medicine, Academic Corporation for the Study of Tropical Pathologies, University of Antioquia, Medellín, Colombia; ^8^Department of Biology, College of Science and Humanities in Al-Kharj, Prince Sattam Bin Abdulaziz University, Al-Kharj, Saudi Arabia; ^9^Department of Zoology and Entomology, Faculty of Science, Helwan University, Cairo, Egypt; ^10^Department of Parasitology, Faculty of Veterinary Medicine, Zagazig University, Zagazig, Egypt; ^11^CIBER Infectious Diseases (CIBERINFEC), Health Institute Carlos III, Madrid, Spain

**Keywords:** epidemiology, genotyping, protists, microsporidia, Zoonoses, transmission

## Abstract

**Introduction:**

Few studies have investigated the occurrence of microeukaryotic gut parasites in dromedary camels in Egypt, and the majority of these investigations are based on microscopic analysis of fecal material.

**Methods:**

Herein, we assessed the occurrence, molecular diversity, and zoonotic potential of protozoan (*Cryptosporidium* spp. and *Giardia duodenalis*) and microsporidian (*Enterocytozoon bieneusi*) pathogens in individual fecal samples (*n* = 102) of dromedary camels with (*n* = 26) and without (*n* = 76) diarrhea from Aswan Governorate, Upper Egypt. Other factors possibly associated with an increased risk of infection (geographical origin, sex, age, and physical condition) were also analyzed. The *SSU* rRNA or ITS genes were targeted by molecular (PCR and Sanger sequencing) techniques for pathogen detection and species identification.

**Results and discussion:**

The most abundant species detected was *G. duodenalis* (3.9%, 4/102; 95% CI: 1.1–9.7), followed by *Cryptosporidium spp*. (2.9%, 3/102; 95% CI: 0.6–8.4). All samples tested negative for the presence of *E. bieneusi*. Sequence analysis data confirmed the presence of zoonotic *C. parvum* (66.7%, 2/3) and cattle-adapted *C. bovis* (33.3%, 1/3). These *Cryptosporidium* isolates, as well as the four *Giardia*-positive isolates, were unable to be amplified at adequate genotyping markers (*Cryptosporidium*: *gp60*; *Giardia*: *gdh, bg*, and *tpi*). Camels younger than 2 years old were significantly more likely to harbor *Cryptosporidium* infections. This connection was not statistically significant, although two of the three cryptosporidiosis cases were detected in camels with diarrhea. The spread of *G. duodenalis* infections was unaffected by any risk variables studied. This is the first report of *C. parvum* and *C. bovis* in Egyptian camels. The finding of zoonotic *C. parvum* has public health implications since camels may function as sources of oocyst pollution in the environment and potentially infect livestock and humans. Although preliminary, this study provides useful baseline data on the epidemiology of diarrhea-causing microeukaryotic parasites in Egypt. Further research is required to confirm and expand our findings in other animal populations and geographical regions of the country.

## 1. Introduction

Globally, *Cryptosporidium* spp., *Giardia duodenalis*, and *Enterocytozoon bieneusi* are among the most prevalent diarrhea-causing enteric parasites in humans and livestock (1–5). These pathogens cause significant morbidity and, in the case of *Cryptosporidium*, mortality in children < 5 years old and immunocompromised persons residing in low-resource settings with little or no access to safe drinking water and sanitation facilities (6, 7). They also pose a threat to public health in middle- and high-income nations (8). These pathogens are transmitted through the fecal–oral route or by direct contact with infected animals or humans. Adult livestock infected with *Cryptosporidium* spp., *G. duodenalis*, and *E. bieneusi* are usually asymptomatic carriers that release varied amounts of (oo)cysts/spores into the surrounding environment and remain a potential source of infection for other animals and humans (9, 10). However, infected neonatal animals may have diarrhea, loss of appetite, lethargy, dehydration, and in some cases, death can occur (11, 12). Importantly, infected neonatal animals can release substantial quantities of instantly infectious (oo)cysts/spores (13, 14), making them important contributors to the (oo)cysts/spore burden in the environment, including surface waters meant for human consumption (15).

Many clinical research facilities in low-income countries rely on microscopy analyses of fecal smears to diagnose enteric parasites (16). Although this method is cheap and easy to perform, it requires well-trained and experienced microscopists, takes time, and lacks diagnostic sensitivity (17). To overcome these limitations, several molecular biological methods for detecting and distinguishing microeukaryotic intestinal parasites have been developed. These include PCR-based genotyping, Sanger sequencing of PCR products, and fluorescence probe-based qPCR techniques (18–20). Molecular methods to improve epidemiological and epidemic studies by allowing researchers to monitor pathogen infection sites, transmission pathways, and virulent genetic variants. For this task, highly sensitive, multi-copy genes, including the small subunit ribosomal RNA (*SSU* rRNA) and the ribosomal internal transcribed spacer (ITS) markers, are widely used (21).

At least 44 *Cryptosporidium* species are considered taxonomically valid (22, 23). Nearly 15 species (*C. andersoni, C. bovis, C. erinacei, C. felis, C. hominis, C. macropodum, C. muris, C. occultus, C. parvum, C. ryanae, C. scrofarum, C. suis, C. tyzzeri, C. ubiquitum*, and *C. xiaoi*) have been reported in domestic ruminants globally, with *C. parvum* the most dominant species, particularly in cattle (3, 20, 24). Seven *Cryptosporidium* species (*C. andersoni, C. bovis, C. hominis, C. muris, C. occultus, C. parvum*, and *C. ubiquitum*), and two genotypes (rat IV and camel) have been identified circulating in camels to date (Table 1).

**Table 1:** Global occurrence and genetic diversity of *Cryptosporidium* spp., *Giardia duodenalis*, and *Enterocytozoon bieneusi* reported in camelids including Bactrian (*Camelus bactrianus*) and dromedary (*Camelus dromedaries*) camels.

**Pathogen**	**Host**	**Country**	**Detection method**	**Frequency % (no. pos./total)**	**Species identified (no.)**	**Genotype (no.)**	**References**
*Cryptosporidium* spp.	DC	Algeria	CM, PCR	5.1 (2/39)	*C. parvum* (2)	If-like (2)	(25)
	DC	Algeria	CM	2.0 (3/149)	*Cryptosporidium* spp. (3)	–	(26)
	DC	Algeria	CM	1.8 (13/717)	*Cryptosporidium* spp. (13)	–	(27)
	DC	Algeria	CM	58.0 (58/100)	*Cryptosporidium* spp. (58)	–	(28)
	DC	Algeria	CM, PCR	10.0 (4/40)	*Cryptosporidium* spp. (4)	ND	(29)
	DC	Australia	PCR	–^a^ (1/1)	*C. parvum* (1)	IIaA17G2R1	(30)
	DC	Azerbaijan	CM	35.7 (65/182)	*C*.*andersoni*^b^ (NA), *C*.*muris*^b^ (NA)	–	(31)
	DC	China	PCR-RFLP	50.0 (2/4)	*C. andersoni* (2)	–	(32)
	DC	Egypt	CM	3.7 (37/1,097)	*Cryptosporidium* spp. (37)	–	(33)
	DC	Egypt	CM	17.5 (14/80)	*Cryptosporidium* spp. (14)	–	(34)
	DC	Egypt	CM	3.8 (4/101)	*Cryptosporidium* spp. (4)	ND	(35)
	DC	Egypt	CM, PCR	19.4 (28/145)	*C. muris* (NA)	–	(36)
	DC	Egypt	CM	24.2 (29/120)	*Cryptosporidium* spp. (29)	–	(37)
	DC	Egypt	PCR-RFLP	5.9 (6/101)	*C. parvum* (2), rat genotype IV (1), and camel genotype (3)	IIaA15G1R1 (1), IIdA19G1 (1)	(38)
	DC	Egypt	CM	8.3 (10/120)	*Cryptosporidium* spp. (10)	–	(39)
	DC	Egypt	CM	20.0 (50/248)	*Cryptosporidium* spp. (50)	–	(40)
	DC	Ethiopia	CM	25.1 (77/307)	*Cryptosporidium* spp. (77)	–	(41)
	DC	Iran	CM	3.3 (13/396)	*Cryptosporidium* spp. (13)	–	(42)
	DC	Iran	CM	1.9 (6/306)	*Cryptosporidium* spp. (6)	–	(43)
	DC	Iran	CM, ELISA	37.9 (39/103)	*Cryptosporidium* spp. (39)	–	(44)
	DC	Iran	CM, ELISA	16.9 (11/65)	*Cryptosporidium* spp. (11)	–	(45)
	DC	Iran	CM, ELISA	4.7 (4/85)	*C. andersoni* (1), *C. muris* (1), and *C. parvum* (2)	–	(46)
	DC	Iran	CM	20.3 (61/300)	*Cryptosporidium* spp. (61)	–	(47)
	DC	Iran	CM	10.0 (17/170)	*Cryptosporidium* spp. (17)	–	(48)
	DC, BC	Iran	CM	81.8 (36/44)	*Cryptosporidium* spp. (36)	–	(49)
	DC	Iran	ELISA	0.5 (1/184)	*C. parvum* (1)	–	(50)
	DC	Iraq	CM	61.0 (61/100)	*Cryptosporidium* spp. (61)	–	(51)
	DC	Iraq	PCR	14.0 (7/50)	*C. parvum* (7)	ND	(52)
	DC	Iraq	CM	55.0 (110/200)	*Cryptosporidium* spp. (110)	–	(53)
	DC	Iraq	CM	37.5 (45/120)	*Cryptosporidium* spp. (45)	–	(54)
	DC	Kuwait	CM	4.0 (10/253)	*Cryptosporidium* spp. (10)	–	(55)
	DC	Saudi Arabia	CM, ELISA	18.4 (9/49); 22.4 (11/49)	*Cryptosporidium* spp. (9–11)	–	(56)
	DC	Saudi Arabia	CM	15.1 (6/33)	*Cryptosporidium* spp. (6)	–	(57)
	DC	Saudi Arabia	ELISA	17.4 (16/92)	*C. parvum* (16)	–	(58)
	BC	China	PCR	–^a^ (1/1)	*C. andersoni* (1)	–	(59)
	BC	China	PCR	–^a^ (1/2)	*C. andersoni* (1)	–	(60)
	NA	China	PCR	15.0 (6/40)	*C. andersoni* (4)*, C. bovis* (2)	ND	(61)
	BC	China	PCR	7.6 (36/476)	*C. andersoni* (24), *C. bovis* (1)*, C. hominis* (1), *C. occultus* (2), *C. parvum* (6), and *C. ubiquitum* (2)	If-like (5), IkA19G1 (1), IIdA15G1 (1), and XIIa (2)	(62)
	BC	China	PCR	–^a^ (2/2)	*C. muris* (2)	–	(63)
	BC	China	PCR	15.0 (6/40)	*Cryptosporidium* spp. (6)	ND	(64)
	BC	China	PCR	NA	*C. muris* (4)	–	(65)
	BC	Czech Republic	PCR	–^a^ (2/2)	*C. muris* (2)	–	(66)
	BC	Czech Republic	PCR	–^a^ (2/2)	*C. andersoni* (2)	–	(67)
	BC	Czech Republic	PCR	–^a^ (1/1)	*C. muris* (1)	–	(68)
	BC	USA	CM	–^a^ (1/1)	*Cryptosporidium* spp. (1)	ND	(69)
	BC	USA	PCR	–^a^ (1/1)	*C. muris* (1)	ND	(70)
	BC, DC	USA	CM	1.3 (1/77)	*Cryptosporidium* spp. (1)	–	(71)
*Giardia duodenalis*	DC	Egypt	CM	5.0 (6/120)	*G. duodenalis* (6)	–	(37)
	DC	Iraq	CM	–^a^ (4/4)	*G. duodenalis* (4)	–	(72)
	DC	Iraq	CM	**24.0 (24**/100)	*G. duodenalis* (24)	–	(51)
	DC	Iraq	CM	20.0 (40/200)	*G. duodenalis* (40)	ND	(53)
	DC	Iraq	CM	4.2 (5/120)	*G. duodenalis* (5)	–	(54)
	DC	Saudi Arabia	CM	–^a^ (7/7)	*G. duodenalis* (7)	–	(73)
	DC, BC	USA	CM	1.3 (1/77)	*G. duodenalis* (1)	–	(71)
	BC	China	PCR	7.5 (3/40)	*G. duodenalis* (3)	A (1), E (2)	(61)
	BC	China	PCR	9.8 (84/852)	*G. duodenalis* (84)	A (14), E (23). A+E (1)	(74)
	BC	China	PCR	7.5 (3/40)	*G. duodenalis* (3)	ND	(64)
	BC	China	PCR	NA	*G. duodenalis* (NA)	A (1) and E (1)	(65)
*Enterocytozoon bieneusi*	DC	Algeria	PCR	20.5 (8/39)	*E. bieneusi* (8)	Camel-2 (2) and Macaque1 (6)	(25)
	BC	China	PCR	30.0 (122/407)	*E. bieneusi* (122)	BEB6 (1), CAM1 (72), CAM2 (8), CAM3 (1), CAM4 (5), CAM5 (1), CAM6 (1), CHG16^c^ (1), CM8 (1), EbpA (5), EbpC (23), Henan-IV (1), O (1), and WL17^d^ (1)	(75)
	BC	China	PCR	45.0 (18/40)	*E. bieneusi* (18)	BEB6 (3), CAM1 (8), and CAM2 (7)	(61)
	BC	China	PCR	NA	*E. bieneusi* (NA)	CD7 (3) and CHS9 (1)	(65)
	BC	China	PCR	45.0 (18/40)	*E. bieneusi* (18)	ND	(64)

ALP, Alpaca; BC, Bactrian camel; CM, Conventional microscopy; DC, Dromedary camel; ELISA, Enzyme-linked immunosorbent assay; NA, not available; ND, not determined; PCR, Polymerase chain reaction; PCR-RFLP, Polymerase chain reaction-restriction fragment length polymorphism.

a Selected positive samples. No prevalence data are available.

b Species-assignment based on morphological differences on the detected *Cryptosporidium* oocysts.

c Synonym of CC1.

d Synonym of EbpC.

*Giardia duodenalis* (syn. *G. intestinalis* and *G. lamblia*) is the only *Giardia* species able to infect domestic ruminants (22, 76). *Giardia duodenalis* is considered a complex cryptic species with eight distinct genetic variants (assemblages A to H), which differ in host distribution and specificity. Assemblages A and B are found in humans and in many other mammals, whereas C and D are found in canids, E in wild and domestic ungulates, F in felids, G in rodents, and H in marine pinnipeds (22, 76). Camels seem to be primarily infected by ungulate-adapted *G. duodenalis* assemblage E; however, zoonotic assemblage A infections have also been reported (Table 1). Remarkably, assemblage E is responsible for 8–100% of cases of human giardiasis documented in Egypt (77–79). More than 600 *E. bieneusi* genotypes have been identified and classified into 11 major phylogenetic groups, of which groups 1 and 2 contain most genotypes with zoonotic potential, and the remaining groups 3–11 include largely host-adapted genotypes associated with specific animal species (80, 81). Today, 15 *E. bieneusi* genotypes have been identified in camels globally, with CAM1 and EbpC accounting for nearly 70% of infections detected (Table 1).

Dromedary camels (*Camelus dromedaries*) have a significant economic, social, and ecological role in nomadic and/or pastoralist communities living in arid or semi-arid regions globally (79). They are natural hosts for a wide range of protists (*Balantioides coli, Blastocystis* sp., *Cryptosporidium* spp., *Enterocytozoon bieneusi, Giardia* spp., *Toxoplasma gondii*, and *Trypanosoma* spp.), helminth (*Echinococcus granulosus, Fasciola hepatica, Schistosoma* spp., and *Trichinella spiralis*), and arthropod (*Linguatula serrata* and *Sarcoptes scabiei*) zoonotic species, representing an often-unrecognized public health threat (27, 82, 83). In addition, infections by some of these pathogens result in significant economic loss due to decreased milk and meat output, diminished fertility, and mortality (84–86).

Several studies in Egypt have looked at the presence of parasite infections, such as *Anaplasma, Babesia, Echinococcus, Sarcocystis, Sarcoptes, Theileria*, and *Trypanosoma* in dromedary camels (87–90). However, evidence on the presence of *Cryptosporidium* spp., *G. duodenalis*, and *E. bieneusi* is even scarcer, with the drawback that most available data come from outdated microscopy-based studies (Table 1). Previous studies have suggested that camels infected with those microeukaryotic parasites might act as potential sources of human cryptosporidiosis, giardiasis, and microsporidiosis (25, 47). To bridge this knowledge gap, this study aims to assess the presence, genetic diversity, and zoonotic potential of *Cryptosporidium* spp., *G. duodenalis*, and *E. bieneusi* in dromedary camels with and without diarrhea in Aswan, the southernmost governorate in Upper Egypt.

## 2. Materials and methods

### 2.1. Study area and sampling

**Figure 1:**
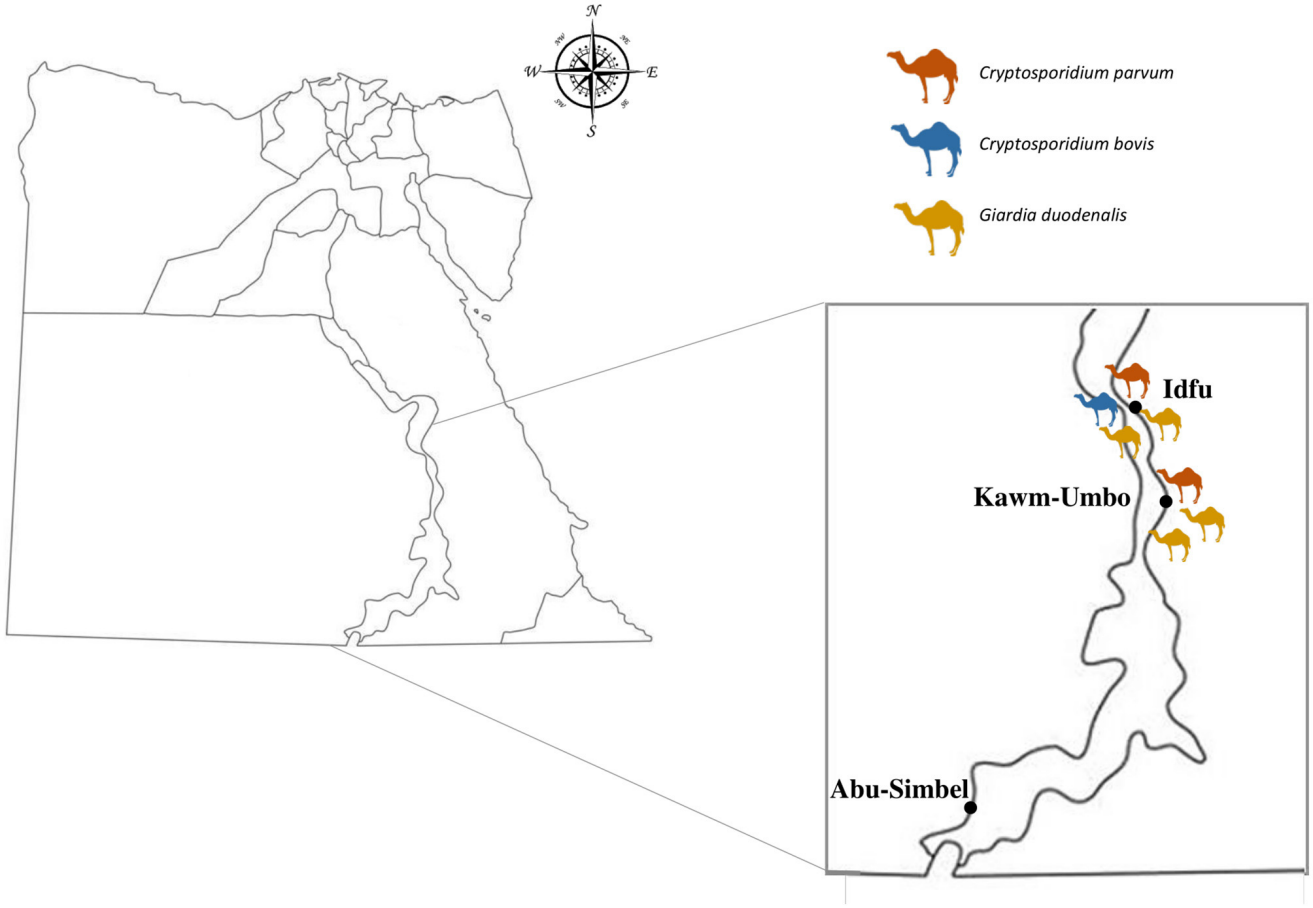
Map of Egypt showing the location of the sampling areas and the distribution of dromedary camels positive to *Cryptosporidium* spp. and *G. duodenalis*.

A total of 102 individual fecal samples from dromedary camels were collected in three geographical areas (Abu Simbel, Edfu, and Kom Ombo) of the Aswan Governorate, Upper Egypt (Figure 1). The calculation of the sample size was performed as described elsewhere (38) based on a 95% confidence level. Fecal samples were collected during the period from August to December 2021. Local farmers were approached and encouraged to participate in the study after their agreement with the study's goals and procedures. Once permission was granted, fecal samples were directly collected from the rectum of the animals and placed into a sterile polystyrene plastic flask containing 70% ethanol as a preservation agent. Basic epidemiological information (geographical origin, sex, age, fecal consistency, and physical condition) was collected at the time of sampling. Animals were reared in an open system under conventional pasture grazing dependent on grazing food including hay and forages. In winter, camels were partly fed on natural grazing, but feeding was complemented by food crops gathered by breeders, and grains may have been added to the diet in certain episodes of production. Out of the 102 samples collected, 26 were diarrheic and 76 formed. Samples were delivered to the Department of Zoonoses, Faculty of Veterinary Medicine (Sohag University, Egypt) and stored at 4°C. Samples were subsequently transferred to the Parasitology Reference and Research Laboratory of the National Center for Microbiology (Majadahonda, Spain) for downstream molecular studies.

### 2.2. DNA extraction and purification

Genomic DNA was isolated from ~200 mg of each fecal sample using the QIAamp DNA Stool Mini Kit (Qiagen, Hilden, Germany) according to the manufacturer's instructions, with the exception that samples combined with InhibitEX buffer were incubated for 10 min at 95°C. DNA samples were extracted and purified before being eluted in 200 μl of PCR-grade water and stored at 4°C until further molecular analysis. A maximum of 18 weeks elapsed between sample collection and DNA extraction.

### 2.3. Molecular detection and characterization of *Cryptosporidium* spp.

The presence of *Cryptosporidium* spp. was assessed using a nested-PCR protocol to amplify a 587-bp fragment of the *SSU* rRNA gene of the parasite (91). Approximately 3 μl of the DNA sample and 0.3 μM of the primer pairs CR-P1/CR-P2 in the primary reaction and CR-P3/CPB-DIAGR in the secondary reaction were used in the amplification procedures (50 μl) (Supplementary Table 1). Both PCR reactions were carried out as follows: one step of 94°C for 3 min, followed by 35 cycles of 94°C for 40 s, 50°C for 40 s, and 72°C for 1 min, concluding with a final extension of 72°C for 10 min.

*Cryptosporidium parvum* isolates were sub-typed by amplifying an 870-bp fragment of the *gp60* locus using a nested PCR (92). Reaction mixtures (50 μl) contained 2–3 μl of template DNA and 0.3 μM of the primer pairs AL-3531/AL-3535 and AL-3532/AL-3534 in the primary and secondary reactions, respectively (Supplementary Table 1). The PCR protocol for the main reaction consisted of an initial step of 94°C for 5 min, followed by 35 cycles of 94°C for 45 s, 59°C for 45 s, and 72°C for 1 min, with a final extension of 72°C for 10 min. The secondary PCR settings were similar to the initial PCR except for the annealing temperature, which was 50°C.

### 2.4. Molecular detection of *Giardia duodenalis*

Detection of *G. duodenalis* DNA was achieved using a real-time PCR (qPCR) method targeting a 62-bp region of the gene codifying the *SSU* rRNA of the parasite (93). Amplification reactions (25 μl) consisted of 3 μl of template DNA, 0.5 μM of each primer Gd-80F and Gd-127R, 0.4 μM of probe (Supplementary Table 1), and 12.5 μl TaqMan^®^ Gene Expression Master Mix (Applied Biosystems, CA, USA). The parasite DNA was detected using a Corbett Rotor Gene^TM^ 6000 real-time PCR system (QIAGEN, Hilden, Germany) with an amplification protocol consisting of an initial hold phase of 2 min at 55°C and 15 min at 95°C followed by 45 cycles of 15 s at 95°C and 1 min at 60°C. Samples with qPCR cycle threshold values <32 were re-analyzed at the glutamate dehydrogenase (*gdh*) (94), β-giardin (*bg*) (95), and triose phosphate isomerase (*tpi*) (96) markers using specific PCR protocols to attempt to identify their assemblages and sub-assemblages.

### 2.5. Molecular detection and characterization of *Enterocytozoon bieneusi*

Detection of *E. bieneusi* was conducted by a nested PCR protocol to amplify the ITS region as well as portions of the flanking large and small subunits of the ribosomal RNA gene, as previously described (97). The outer EBITS3/EBTIS4 and inner EBITS1/EBITS2.4 primer sets (Supplementary Table 1) were used to generate PCR products of 435 and 390 bp, respectively. The main PCR was cycled at 94°C for 3 min, followed by 35 cycles of amplification (denaturation at 94°C for 30 s, annealing at 57°C for 30 s, and elongation at 72°C for 40 s), with a final extension at 72°C for 10 min. Conditions for the secondary PCR were identical to the primary PCR, except that only 30 cycles were performed at an annealing temperature of 55°C.

### 2.6. PCR and gel electrophoresis standard procedures

All of the aforementioned direct and nested PCR protocols were conducted on a 2720 Thermal Cycler (Applied Biosystems). Reaction mixes always included 2.5 units of MyTAQ^TM^ DNA polymerase (Bioline GmbH, Luckenwalde, Germany), and 5–10 μl of MyTAQ^TM^ Reaction Buffer with 5 mM dNTPs and 15 mM MgCl_2_. For each parasite species studied, laboratory-confirmed positive and negative DNA samples of human and animal origin were routinely used as controls and included in each round of PCR. PCR amplicons were visualized on 1.5% D5 agarose gels (Conda, Madrid, Spain) stained with Pronasafe (Conda) nucleic acid staining solutions. A 100-bp DNA ladder (Boehringer Mannheim GmbH, Baden-Wurttemberg, Germany) was used to size the obtained amplicons.

### 2.7. Sequence analyses

Positive-PCR products of the expected size were directly sequenced in both directions using appropriate internal primer sets (Supplementary Table 1). DNA sequencing was conducted by capillary electrophoresis using the BigDye^®^ Terminator chemistry (Applied Biosystems) on an ABI PRISM 3130 automated DNA sequencer. Generated DNA consensus sequences were aligned to appropriate reference sequences using MEGA6 (98) for species confirmation and genotype identification. The sequences obtained in this study have been deposited in GenBank under accession numbers OP365100 (*C. bovis*) and OP365101–OP365102 (*C. parvum*).

### 2.8. Statistical analyses

Fisher's exact tests were used to assess the relationships between parasitic infections and the different independent factors addressed in the study (geographical origin, sex, age, fecal consistency, and physical condition). A *P-*value of < 0.05 was considered statistically significant. Analyses were conducted using the statistical package SPSS version 25 (IBM Corporation, Armonk, NY, USA).

## 3. Results

### 3.1. Occurrence of the parasites

*Giardia duodenalis* was the most prevalent species found (3.9%, 4/102; 95% CI: 1.1–9.7), followed by *Cryptosporidium* spp. (2.9%, 3/102; 95% CI: 0.6–8.4). In contrast, *E. bieneusi* DNA was not detected in the dromedary camel population under investigation. The distribution of the *Cryptosporidium* and *G. duodenalis* infections according to the variables considered in the study is shown in Table 2. *Cryptosporidium* infections were detected in male animals younger than 5 years age from Edfu and Kom Ombo localities. Two of the three infections were detected in animals that had diarrhea. One of the three cryptosporidiosis-infected animals had emaciation, weakness, and roughened skin. *Giardia* infections were also detected in male dromedary camels only from Edfu and Kom Ombo localities. In contrast to *Cryptosporidium*, all *Giardia* infections were found in animals older than 5 years of age, primarily without diarrhea and in good physical condition. None of the three intestinal protist species proved positive in the dromedary camels sampled at Abu Simbel.

**Table 2:** Distribution of *Cryptosporidium* spp. and *Giardia duodenalis* infections according to geographical origin, sex, age, fecal consistency, and physical condition of examined camels (*n* = 102).

**Variable**	**Total (** ***n*** **)**	***Cryptosporidium*** **spp**.			***Giardia duodenalis***		
		**Infected (** ***n*** **)**	**%**	***P*** **-value**	**Infected (** ***n*** **)**	**%**	***P*** **-value**
**Geographical origin**							
Abu-Simbel	35	0	0	0.53	0	0	0.46
Idfu	35	2	5.7		2	5.7	
Kawm-Umbo	32	1	3.1		2	6.2	
**Sex**							
Male	95	3	3.2	1	4	4.2	1
Female	7	0	0		0	0	
**Age (yrs.)**							
≤ 2	13	2	15.4	0.01^*^	0	0	1
2–5	15	1	6.7		0	0	
≥5	74	0	0		4	5.4	
**Diarrhea**							
Yes	26	2	7.7	0.17	1	3.8	1
No	76	1	1.3		3	4	
**Physical condition**							
Normal	84	2	2.4	0.45	4	4.8	1
Emaciated	18	1	5.6		0	0	

Statistically significant values are highlighted in bold with a star (Fisher exact test was used).

NS, not statistically significant at the Fisher exact test.

* P-value < 0.05: statistically significant.

### 3.2. Risk association analyses

Dromedary camels younger than 2 years were significantly more likely to be infected by *Cryptosporidium* spp. than animals of older age (*P* < 0.05). None of the remaining variables were associated with an increased risk of infection by *Cryptosporidium* spp. or *G. duodenalis*.

### 3.3. Molecular data

The results of the *Cryptosporidium* sequencing analysis generated in the present study are summarized in Table 3. One of the three *Cryptosporidium*-positive samples was identified as cattle-adapted *C. bovis*, showing 100% identity with a stretch of 455 bp from position 315–770 of reference sequence AY741305. The remaining two samples were recognized as zoonotic *C. parvum*, and their sequences varied from reference sequence AF112571 by four to five single nucleotide polymorphisms (SNPs), including a TAAT deletion at positions 686–689 of AF112571. During the visual assessment of chromatograms, no ambiguous positions in the form of double peaks were found. Attempt to amplify the *C. parvum* isolates at the *gp60* locus failed, so the subtype family of the parasite remained unknown.

**Table 3:** Frequency and molecular diversity of *Cryptosporidium* spp. identified in camels in the present study.

**Species**	**No. isolates**	**Locus**	**Reference sequence**	**Stretch**	**Single nucleotide polymorphisms**	**GenBank ID**
*C. bovis*	1	*SSU* rRNA	AY741305	315–770	None	OP365100
*C. parvum*	1	*SSU* rRNA	AF112571	544–983	A646G, T649G, 686_689delTAAT, and T693A	OP365101
*C. parvum*	1	*SSU* rRNA	AF112571	527–1,030	A646G, T649G, 686_689delTAAT, T693A, and T972A	OP365102

del, Deletion; *SSU* rRNA, Small subunit ribosomal RNA.

All four *G. duodenalis*-positive isolates yielded C_T_ values >32 (median: 35.9; range: 32.8–38.5) at qPCR, indicating a relatively low quantity of parasite DNA in the original samples. None of these samples could be amplified at the *gdh, bg*, and *tpi* loci.

## 4. Discussion

This study adds to the body of knowledge about the occurrence and genetic diversity of the diarrhea-causing intestinal protists *Cryptosporidium* spp., *G. duodenalis*, and *E. bieneusi* in Egyptian dromedary camels. The main strength of the survey is the use of PCR and Sanger sequencing technologies, allowing for accurate detection, differentiation, and characterization of the investigated pathogens. The survey is also relevant because (i) it focuses on a host species (dromedary camel) for which parasite epidemiological data are particularly scarce in Egypt, (ii) it demonstrates that dromedary camels can act as the potential source of human cryptosporidiosis caused by *C. parvum*, and (iii) information gathered is useful for developing proper intervention and control strategies against oral–fecal transmitted diseases, including cryptosporidiosis and giardiasis (79, 99).

*Cryptosporidium* infections were detected in 3% of the investigated dromedary camels. Surprisingly, its incidence percentage was lower (4–24%) than those detected by conventional microscopy in other Egyptian camel populations (33–37, 39, 40). However, a slightly superior rate of 6% was reported in a similar study conducted using PCR-RFLP (38). These disparities between microscopy and PCR data might be attributed to fundamental epidemiological (infection pressure and geographical area) and host (age and immunological state) differences among the camel populations surveyed. However, unwanted false-positive results are prevalent during microscope investigation and might lead to overestimated prevalence rates (100). Similar highly variable *Cryptosporidium* prevalences have been observed by conventional microscopy or ELISA techniques in various Middle Eastern countries, including Iran (2–100%), Iraq (7–100%), and Saudi Arabia (15–22%; see Table 1). Our genotyping data revealed the presence of two *Cryptosporidium* species, including *C. parvum* (in two animals presenting with diarrhea) and *C. bovis* (in an asymptomatic animal). *Cryptosporidium* infections have been previously reported in diarrheic dromedary camels in Algeria (27) and Iran (48), whereas *C. parvum* has already been described in Egyptian dromedary camels (38); this is the first report of cattle-adapted *C. bovis* in this host species in the country and the third report globally after the description of the parasite in Bactrian camels in China (61, 62). In Egypt, previous research has revealed the occurrence of *C. bovis* in cattle and buffalo populations (101–105). These findings show that *C. bovis* cross-species transmission is likely in areas where different domestic ruminant species share habitat. Although the two dromedary camels infected with this *Cryptosporidium* species manifested diarrhea, we were unable to amplify the two *C. parvum* isolates at the *gp60* locus. The lack of diagnostic data for viral or bacterial agents was an obstacle to unambiguously linking the occurrence of diarrhea with a given enteric pathogen. In this regard, light *C. parvum* infections associated with modest oocyst shedding might explain the amplification failure at the single copy *gp60* gene, a marker known for its limited diagnostic sensitivity (21). Notably, *C. parvum gp60* genotype families IIa and IId have been found in Egyptian dromedary camels (38). It should be stressed that *C. parvum* is regarded as a common zoonotic *Cryptosporidium* species with loose host specificity and worldwide distribution, whereas human cases of cryptosporidiosis caused by *C. bovis* are sporadically reported globally (22, 23). Therefore, our molecular data support the potential zoonotic spread of those *Cryptosporidium* species between infected dromedary camels and humans.

In the present study, *G. duodenalis* was the predominant (4%) protozoan parasite found among the examined camel population. Conventional microscopy revealed a fairly comparable *G. duodenalis* infection rate of 5% in the sole prior investigation undertaken on this host species in Egypt (37). Epidemiological information on camel populations in other Middle Eastern countries is also scarce and completely absent in African countries other than Egypt. Prevalence rates of 4–24% have been documented in Iraq (51, 53, 54). The parasite is also known to be circulating at an unknown infection rate in dromedary camels in Saudi Arabia (73). All the previously mentioned studies were based on conventional microscopy, so information on the *G. duodenalis* assemblages and sub-assemblages causing the infections is also lacking. It is noteworthy that *G. duodenalis* has been detected at occurrence rates of 7–10% in Chinese Bactrian camels by PCR (61, 64, 65, 74). All these infections were caused primarily by ungulate-adapted *G. duodenalis* assemblage E and, to a lesser extent, by zoonotic *G. duodenalis* assemblage A (see Table 1). In our study, the four *G. duodenalis*-positive samples (three in asymptomatic animals and one in a diarrheic animal) yielded high C_T_ values (>32) at qPCR and impeded the completion of genotyping analyses at appropriate genetic markers, including the genes encoding for the glutamate dehydrogenase (*gdh*), beta-giardin (*bg*), and triosephosphate isomerase (*tpi*) proteins of the parasite. As in the case of the *Cryptosporidium gp60* locus, the *Giardia gdh, bg*, and *tpi* loci are single-copy genes with limited diagnostic sensitivities, making them unsuitable for amplifying samples with a small amount of parasitic DNA. The high C_T_ values obtained at qPCR are also indicative of light infections, compatible with the absence of gastrointestinal manifestations (diarrhea) in most *Giardia*-positive dromedary camels. The lack of genotyping data at the assemblage and sub-assemblage levels does not allow us to fully assess the zoonotic implications of our findings. More research should be conducted to ascertain the genetic diversity of *G. duodenalis* infections in camels and their role as potential sources of human giardiasis.

No DNA of the microsporidia *E. bieneusi* could be detected in any of the fecal DNA samples analyzed in the present study, suggesting that dromedary camels are not relevant hosts in the transmission of this pathogen in Egypt. Very few epidemiological studies have attempted to investigate the occurrence and genetic diversity of *E. bieneusi* in camels globally. In the only survey conducted in Africa to date, a PCR prevalence rate of 20% was estimated in Algerian dromedary camels (25). In that study, two *E. bieneusi* genotypes were detected, including Camel-2 and Macaque1. More information is available from Bactrian camels in China, where *E. bieneusi* seems to be a common finding with infection rates in the range of 30–45% (61, 64, 65, 75). Most of the infections detected were caused by camel-adapted *E. bieneusi* genotypes, including CAM1 to CAM6, but the presence of genotypes such as BEB6, EbpA, EbpC, and O (all four members of phylogenetic Groups 1 and 2, including zoonotic genetic variants of the parasite) indicate that Bactrian camels can serve as potential sources of *E. bieneusi* infections to humans (77, 78). It should be noted that, in Egypt, *E. bieneusi* has been previously detected in immunosuppressed patients with and without diarrhea (106, 107), in children attending day-care centers (108), and in domestic animals including cattle, buffaloes, rabbits, sheep, goats, cats, and dogs (109). These data highlight the need to investigate the role of other animal host species (including dromedary camels) as potential sources of human microsporidiosis by *E. bieneusi* in the country.

Regarding the analysis of variables potentially associated with an increased risk of infection by enteric protists, dromedary camels younger than 2 years of age were more likely to be infected by *Cryptosporidium* spp., this being the only statistically significant association found in the present study. This result is consistent with those obtained in a study that found greater *Cryptosporidium* infection rates in 1-year-old camel calves than in older animals in Iran (48). Discrepant results have been reported in other surveys. For instance, *Cryptosporidium* infections were more frequently identified in camels in the age groups of 1–4 years in Algeria (27) and 3–6 years in Iraq (51). A third study that was conducted in Iran revealed no significant associations between camel age and *Cryptosporidium* infection status (44). Although not statistically significant, all dromedary camels sampled at Abu Simbel tested negative for *Cryptosporidium* spp., *G. duodenalis*, and *E. bieneusi*, suggesting that environmental (e.g., geographical area of origin and local climatic conditions) and biological (e.g., host age and immunological status) conditions and management practices (e.g., contact with other livestock) might play a role in the occurrence and distribution of these pathogens. Taken into account, most of the studied animals were reared in resource-poor settings, including water and food sources, which, together with the management practices, affect the occurrence of the reported pathogens. A lack of access to safe drinking water and poor sanitation and hygiene practices were identified as potential factors linked with a higher risk of developing diarrhoeal illness (15). In relation to feeding habitat, several previous studies have revealed an obvious association between the occurrence of various parasites in camels and grazing performance, including bushes and grasses. In this respect, logging of shrubs, bushes, and trees for rain-fed production systems might enhance the probability of harvesting the ova and/or larvae from pasture (110). Given the above findings, our study pointed out that the application of strict control and hygienic measures represented by providing clean drinking water, improvement of sanitation and hygiene practices are mandatory preventive strategies to control these zoonotic pathogens. Furthermore, regular administration of antiparasitic drugs and treatment of infected camels in the studied area stand as major control measures for the infection and should be adopted, together with the strict quarantine of imported animals from neighboring regions.

Some design and methodological limitations might have biased the accuracy of the results obtained in the present study and should be considered when interpreting them. First, the smaller sample size may have led to underestimating true prevalence rates and lowered the power of the statistical analyses conducted. Second, the transversal nature of the study might not be adequate to capture potential temporal/seasonal variations in parasite occurrence. Third, the animal population under study was mainly composed of adult animals, which are less likely to be infected by the diseases studied. Fourth, suboptimal fecal sample storage and transportation conditions might have altered the quantity and quality of the DNA used for diagnostic and genotyping purposes. Fifth, the lack of genotyping data for some of the protist species investigated (e.g., *G. duodenalis*) made it difficult to fully analyze the epidemiological and zoonotic implications of our findings.

## 5. Conclusion

This is one of the very few molecular-based epidemiological studies aiming at investigating the presence and molecular diversity of diarrhea-causing enteric protist parasites in dromedary camels in African countries, including Egypt. *Cryptosporidium* spp. and *G. duodenalis* were identified at low (< 5%) infection rates. Sequence analyses revealed the presence of two *Cryptosporidium* species, including zoonotic *C. parvum* and cattle-adapted *C. bovis*. This is the first report of *C. bovis* in dromedary camels globally. The presence of *C. parvum* implies that dromedary camels play a role in the transmission of this *Cryptosporidium* species and can serve as potential sources of human cryptosporidiosis. Implementation of stricter hygienic measures and awareness raising are recommended to minimize the zoonotic hazard of camel pathogens to people in contact with these animals or their manure. Improving water and food resources in the studied area seems mandatory to reduce the transmission of infection by these zoonotic pathogens. Further research is warranted to corroborate and expand these preliminary findings in larger camel populations and other animal species in Upper Egypt.

## Data availability statement

The original contributions presented in the study are included in the article/Supplementary material, further inquiries can be directed to the corresponding authors.

## Ethics statement

The animal study was reviewed and approved by the Research Ethics Committee of the Faculty of Veterinary Medicine, Sohag University (Egypt) on 01.12.2019. Written informed consent was obtained from the owners for the participation of their animals in this study.

## Author contributions

EE, SA, AS, AG, ML, BA, and EH collected the samples. EE, PK, AD, CH-C, and BB conducted laboratory experiments. PK and AD conducted sequence analyses. EE conducted statistical analyses. SA and ML secured the funding for conducting sampling and experimental work. EE, DG-B, and DC designed and supervised the experiments. EE and DC writing—original draft preparation. EE, SA, AG, AS, AD, DG-B, and DC writing—review and editing. The final version was read and approved by all authors.
