# Supplementary material for: Molecular detection and characterization of Cryptosporidium spp., Giardia duodenalis, and Enterocytozoon bieneusi infections in dromedary camels (Camelus dromedaries) in Egypt
Source: Front Vet Sci. 2023 Apr 20;10:1139388. doi: 10.3389/fvets.2023.1139388 (PMC10157078; doi:10.3389/fvets.2023.1139388)
Supplement: Supplementary file 1 [file Data_Sheet_1.PDF]

Supplementary Table 1. Oligonucleotides used for the molecular identification and/or characterization of the protist and microsporidia pathogens investigated in this study.

Target organism	Locus	Oligonucleotide	Sequence (5´–3´)	Reference
Cryptosporidium spp.	*ssu* rRNA	CR-P1	CAGGGAGGTAGTGACAAGAA	(91)
		CR-P2	TCAGCCTTGCGACCATACTC	(91)
		CR-P3	ATTGGAGGGCAAGTCTGGTG	(91)
		CPB-DIAGR	TAAGGTGCTGAAGGAGTAAGG	(91)
	gp60	AL 3531	ATAGTCTCCGCTGTATTC	(92)
		AL 3535	GGAAGGAACGATGTATCT	(92)
		AL 3532	TCCGCTGTATTCTCAGCC	(92)
		AL 3534	GCAGAGGAACCAGCATC	(92)
Giardia duodenalis	*ssu* rRNA	Probe	FAM–CCCGCGGCGGTCCCTGCTAG–BHQ1	(93)
		Gd-80F	GACGGCTCAGGACAACGGTT	(93)
		Gd-127R	TTGCCAGCGGTGTCCG	(93)
Enterocytozoon bieneusi	ITS	EBITS3	GGTCATAGGGATGAAGAG	(97)
		EBITS4	TTCGAGTTCTTTCGCGCTC	(97)
		EBITS1	GCTCTGAATATCTATGGCT	(97)
		EBITS2.4	ATCGCCGACGGATCCAAGTG	(97)

gp60: 60Kda glycoprotein; ITS: Internal transcribed spacer; *ssu* rRNA: Small subunit ribosomal RNA.
